# Metergoline Shares Properties with Atypical Antipsychotic Drugs Identified by Gene Expression Signature Screen

**DOI:** 10.1007/s12640-023-00673-0

**Published:** 2023-11-03

**Authors:** Chiara C Bortolasci, Emily J Jaehne, Damián Hernández, Briana Spolding, Timothy Connor, Bruna Panizzutti, Olivia M Dean, Tamsyn M Crowley, Alison R Yung, Laura Gray, Jee Hyun Kim, Maarten van den Buuse, Michael Berk, Ken Walder

**Affiliations:** 1 The Institute for Mental and Physical Health and Clinical Translation, Deakin University, Geelong, Australia; 2 School of Psychology and Public Health, La Trobe University, Bundoora, Australia; 3 Florey Institute for Neuroscience and Mental Health, The University of Melbourne, Melbourne, Australia; 4 School of Health Sciences, University of Manchester, Manchester, UK; 5 Centre for Youth Mental Health, University of Melbourne, Parkville, Australia

**Keywords:** Psychiatric disorders, Psychosis, Treatment, Psychiatry, Neuroscience, Drug discovery

## Abstract

Novel approaches are required to find new treatments for schizophrenia and other neuropsychiatric disorders. This study utilised a combination of in vitro transcriptomics and in silico analysis with the BROAD Institute’s Connectivity Map to identify drugs that can be repurposed to treat psychiatric disorders. Human neuronal (NT2-N) cells were treated with a combination of atypical antipsychotic drugs commonly used to treat psychiatric disorders (such as schizophrenia, bipolar disorder, and major depressive disorder), and differential gene expression was analysed. Biological pathways with an increased gene expression included circadian rhythm and vascular endothelial growth factor signalling, while the adherens junction and cell cycle pathways were transcriptionally downregulated. The Connectivity Map (CMap) analysis screen highlighted drugs that affect global gene expression in a similar manner to these psychiatric disorder treatments, including several other antipsychotic drugs, confirming the utility of this approach. The CMap screen specifically identified metergoline, an ergot alkaloid currently used to treat seasonal affective disorder, as a drug of interest. In mice, metergoline dose-dependently reduced MK-801- or methamphetamine-induced locomotor hyperactivity confirming the potential of metergoline to treat positive symptoms of schizophrenia in an animal model. Metergoline had no effects on prepulse inhibition deficits induced by MK-801 or methamphetamine. Taken together, metergoline appears a promising drug for further studies to be repurposed as a treatment for schizophrenia and possibly other psychiatric disorders.

## Introduction

Neuropsychiatric disorders such as schizophrenia have a significant negative effect on the quality of life of affected individuals and their families. It is estimated that more than 650 million people suffer from neuropsychiatric disorders worldwide (World Health Organization 2006). However, the pathophysiology of these disorders is still not fully understood and the discovery of novel treatments has stalled. One of the major limitations of the progression of new treatments is the high complexity of brain function and the lack of human brain tissue access for analysis.

In complex diseases with poorly understood pathophysiology, it is imperative to use novel approaches to discover new treatment options (Berk and Nierenberg 2015; Kidnapillai et al. 2020). The aim of this study was to identify potential new drug candidates that affect gene expression in a similar manner to the common effects of widely used antipsychotics to treat neuropsychiatric disorders such as schizophrenia. These drugs typically work well in a small proportion of patients but do not have beneficial effects in all patients. Therefore, we measured the transcriptomic effects in cultured human neuronal cells treated with a combination of currently prescribed atypical antipsychotic drugs that are thought to be mechanistically diverse. A combination of drugs was used as we did not want to simply find the drug most similar to existing treatment but to mimic the many biological pathways regulated to a different extent by a range of antipsychotic drugs. Next, we used these data and a systematic approach to associate candidate drugs to the enriched genetic pathways from the treatment of atypical antipsychotic drugs on neuronal cells, as an in silico screen for potential drugs for the treatment of neuropsychiatric disorders. This approach identified potential drugs including other antipsychotic drugs, confirming the validity of this approach. Specifically, metergoline, an ergot alkaloid currently used to treat seasonal affective disorder, was identified as a drug of interest.

The identified candidate drug, metergoline, was then tested in mice using a psychosis-like behavioural test commonly used as a positive symptom model of schizophrenia. Treatment of mice with metergoline dose-dependently reduced MK-801- or methamphetamine-induced locomotor hyperactivity with no effect on prepulse inhibition deficits induced by MK-801 or methamphetamine. Our results highlight metergoline as a potential candidate drug for the treatment of schizophrenia and other psychiatric disorders.

## Materials and Methods

### In Vitro

#### Cell Culture and RNA Isolation

Ntera2 human teratocarcinoma cells (NT2, ATCC) were cultured and differentiated into neuronal-like (NT2-N) cells as described previously (Pleasure et al. 1992). Briefly, NT2 cells were treated with 10 µM retinoic acid (Sigma-Aldrich) for 28 days to induce differentiation into NT2-N cells. Differentiated NT2-N cells were treated for 24 h with either the combination of atypical antipsychotic drugs (10 µM amisulpride, 0.1 µM aripiprazole, 10 µM clozapine or 0.1 µM risperidone) or vehicle (0.1% DMSO) as control with 20 independent experiments per group (n = 20/group). Drug doses were determined empirically such that no single drug dominated the overall effects on gene expression (data not shown). RNA was extracted from harvested NT2-N cells using TRIzol reagent (Thermo Fisher Scientific) and purified using RNeasy mini kit (Qiagen). NanoDrop 1000 instrument (Thermo Fisher Scientific) determined RNA quantity, and Agilent 2100 Bioanalyser (Agilent) determined RNA quality.

#### Next-Generation Sequencing

RNA sequencing libraries from 1 mg of total RNA were prepared (Illumina TruSeq RNA Sample Preparation Kits) and sequencing was performed using an Illumina HiSeq platform (HiSeq 2500 rapid 50bpSE; one flow cell). Raw sequencing data were aligned to the reference genome (Bowtie 2/TopHat 2) (Kim et al. 2013), low count genes were removed, and the data normalised using the weighted trimmed mean of *M* values. Genes that were differentially expressed between the drug combination- and vehicle-treated cells were identified using edgeR in R (Robinson et al. 2010).

## In Silico

### Connectivity Map (CMap)

The top 50 genes with the best evidence for increased or decreased expression due to antipsychotic drug combination compared to the vehicle were identified from the edgeR output and designated as the “gene expression signature” for this study. For each of these genes, a corresponding probe recognised by CMap was identified (HG-U133A array) and used to interrogate the CMap database (Lamb et al. 2006).

### In Vivo

#### Animals

C57Bl/6 mice were purchased from Animal Resources Centre (WA, Australia). Ninety-six mice were tested in total (48 males and 48 females). All mice were housed in groups of 4 in individually ventilated cages (Tecniplast, Buguggiate, Italy) under a 12-h light-dark cycle (lights on: 07:00) with food and water available *ad libitum*. Housing and testing rooms were 21 ± 2 °C. All testing was conducted between 08:00 and 16:00. All procedures were approved by the La Trobe University Animal Ethics Committee in accordance with the Australian Code of Practice for the Care and Use of Animals for Scientific Purposes (National Health and Medical Research Council of Australia).

#### Drug Treatment and Experimental Procedure

Metergoline and MK-801 were purchased from Sigma-Aldrich (USA) and methamphetamine was supplied by the National Measurement Institute (NSW, Australia). Metergoline was dissolved in vehicle (10% DMSO in PBS) and was intraperitoneally (i.p.) injected at 0.3 or 1 mg/kg for locomotor tests (due to previously observed sedative effects (Varty and Higgins 1995; Glass et al. 2012)) and 1 or 3 mg/kg for PPI (Varty and Higgins 1995; Cheetham and Heal 1993) 30 min prior to either methamphetamine (3 mg/kg) or MK-801 (0.25 mg/kg) injection (i.p. dissolved in 0.9% sterile saline). All injection volumes were 5 ml/kg.

Mice were allocated into 4 different assessments to test for metergoline effects on locomotor hyperactivity or prepulse inhibition (PPI) following either methamphetamine or MK-801. Twelve males and twelve females were allocated per assessment (*n* = 24/assessment). In each of the assessments, every mouse underwent six sessions of behavioural testing (i.e., 6 within-subjects conditions). Specifically, the six sessions involved the administration of (1) vehicle + saline, (2) metergoline low dose + saline, (3) metergoline high dose + saline, (4) vehicle + methamphetamine or MK-801,(5) metergoline low dose + methamphetamine or MK-801, and (6) metergoline high dose + methamphetamine or MK-801, given in a pseudorandomised order. Mice were given at least 3 or 4 days’ rest between every behavioural test to allow wash out of drugs used.

#### Locomotor Hyperactivity

Mice were placed into automated photocell arenas (Med Associates, VT, USA), 27 × 27cm with 40 cm walls (16 × 16 array of photobeam sensors). Mice were first placed in the arenas for 30 min, then were removed briefly and injected with metergoline or vehicle, then placed back into the chambers for 30 min, then they were again removed briefly for saline, methamphetamine or MK-801 injection, then placed back into the arena for 2 more hours.

#### Prepulse Inhibition of Acoustic Startle

PPI was assessed using automated SR-Lab startle chambers (San Diego Instruments, CA, USA). Mice were placed in individual plexiglass cylinders (5 cm diameter) and the test session comprised 104 stimulus trials as previously described (Manning and van den Buuse 2013; Notaras et al. 2017). Briefly, test sessions consisted of four blocks of 8 × 115 dB startle alone pulses of 40 ms. Two blocks included prepulse trials consisting of a single 115 dB pulse preceded either 100 or 30 ms by a 20 ms prepulse of 2, 4, 8 or 16 dB over background maintained at 65 dB. PPI was quantified as the difference between startle responses during prepulse-pulse and pulse alone trials and expressed as a percentage of pulse alone responses. Mice showed low and variable PPI at 30 ms inter-stimulus interval; therefore, analysis focussed on the 100 ms inter-stimulus interval. Following metergoline or vehicle and methamphetamine or MK-801 injection, mice were placed into startle chambers immediately after methamphetamine or 15 min after MK-801 injection.

## Data Analysis

Analysis of variance (ANOVA) with repeated measures was used to assess drug effects on behaviours (IBM SPSS Statistics v26, or GraphPad Prism 9.0). Within-group factors were: treatment (vehicle, metergoline low dose, metergoline high dose) x induction (saline vs. methamphetamine or MK-801), and the between-group factor was sex (male vs. female). Significant main effects and interactions were further explored using Tukey post hoc multiple comparisons.

## Results

### Next Generation Sequencing

Next-generation sequencing generated 415 million raw reads (10.4 million reads averaged per sample) with 21,143 million total bases (529 million bases averaged per sample) from 40 libraries of NT2-N cells treated with the schizophrenia drug combination or vehicle control. After filtering and trimming, data were obtained for 11,355 elements. Of these, 1555 elements exhibited differential expression following the drug cocktail treatment (adjusted *p* < 0.05), with 905 having higher and 650 having lower expression.

### GSEA

The gene expression dataset was submitted to the BROAD Institute portal for gene set enrichment analysis. Genes that were differentially expressed following the drug treatment were enriched for several pathways (Table 1).

**Table 1:** Pathways enriched for genes with higher or lower expression following drug treatment of NT2-N cells (relative to vehicle)

Kegg pathway	Size	NES	NOM *p*-val	FDR *q*-val
Higher expression				
Circadian rhythm mammal	13	2.65	<0.001	<0.001
VEGF signalling pathway	62	2.12	<0.001	<0.001
Long term potentiation	62	1.85	<0.001	0.037
Lower expression				
Adherens junction	72	−2.32	<0.001	<0.001
Cell cycle	120	−2.15	<0.001	<0.001
Proteasome	41	−1.96	<0.001	0.0090
Tight junction	110	−1.93	<0.001	0.0091
Nucleotide excision repair	42	−1.87	<0.001	0.014
Pancreatic cancer	65	−1.81	<0.001	0.016
Endometrial cancer	50	−1.79	<0.001	0.020
Renal cell carcinoma	66	−1.76	<0.001	0.022
Arginine and proline metabolism	45	−1.74	<0.001	0.027
Tryptophan metabolism	31	−1.70	<0.001	0.034
P53 signalling pathway	65	−1.69	<0.001	0.035
Hypertrophic cardiomyopathy	64	−1.66	<0.001	0.043

Size is the number of genes in the pathway

*NES* normalised enrichment score, *VEGF* vascular endothelial growth factor

### CMap

CMap is a gene expression-based drug development system that incorporates genes, drugs and diseases. The CMap analysis was conducted using the 50 genes with best evidence of higher expression and 50 genes with lower expression following combination antipsychotic drug treatment (Table 2) of NT2-N cells. The CMap analysis results on drug matches are shown in Table 3. Candidate drugs were further analysed and some were excluded from further investigation if: (a) unsuitable for long term use; (b) not novel (including antipsychotic drugs already in use for the treatment of schizophrenia); (c) having unacceptable side effects (including cytotoxic drugs used in oncology and other drugs with a black box warning); (d) not approved for use in humans; and (e) poor or no oral bioavailability. After exclusion, metergoline was identified as the best drug of interest.

**Table 2:** Genes included in the CMap analysis

A				B			
Name	logFC	adj.p.value	totreads	Name	logFC	adj.p.value	totreads
NUPR1	1.32	1.68E-170	4140	ANKRD1	−1.88	8.55E-258	3490
CDKN1A	0.57	1.37E-134	19400	TUBA1A	−0.64	3.94E-193	29950
SQSTM1	0.53	1.34E-96	14322	ACTB	−0.78	1.08E-192	39965
SCD	0.68	3.21E-95	11468	TUBA1B	−0.71	3.02E-191	17663
MMP1	1.84	4.51E-89	1217	NPPB	−2.57	1.15E-165	1667
FADS2	0.59	1.49E-73	8835	TUBA1C	−0.70	5.62E-163	14554
ATF4	0.72	1.46E-65	4977	IGFBP3	−1.55	2.96E-130	2459
CD82	0.70	1.15E-52	4209	TPM1	−0.57	3.74E-125	34598
SAT1	0.67	8.79E-52	12583	TUBB	−0.54	1.36E-121	20045
ITM2C	0.43	2.61E-50	13077	ANXA2	−0.46	1.05E-102	35499
SPP1	0.48	8.54E-46	8116	ACTG1	−0.56	2.67E-99	27884
FASN	0.52	8.64E-40	5732	ANXA1	−0.53	5.44E-91	13872
TKT	0.39	7.96E-38	10851	CCND1	−0.74	6.19E-78	5650
EVA1B	0.54	5.19E-37	5026	SLC2A3	−0.63	1.64E-65	6646
HIST3H2A	0.64	2.96E-35	3416	FSTL1	−0.45	7.48E-55	12539
HIST2H2AA3	0.59	2.98E-33	3791	MEST	−0.58	8.24E-55	6651
HIST2H2AA4	0.59	2.98E-33	3791	TUBB4B	−0.59	6.81E-51	5737
PTX3	0.81	1.46E-32	1978	CALD1	−0.78	2.54E-50	3323
DBI	0.35	1.74E-32	11321	CTGF	−0.96	1.53E-49	2312
C5orf45	0.71	2.98E-32	2541	CALM2	−0.45	5.64E-49	14444
FTL	0.34	7.52E-31	165906	S100A10	−0.33	2.48E-43	18025
DHCR7	0.43	2.30E-30	6588	PLK2	−0.78	5.22E-43	2875
VWA5A	0.82	1.20E-29	1746	PFN2	−0.38	4.60E-42	12473
UBC	0.25	1.37E-29	39446	CYR61	−1.43	2.88E-39	837
MT2A	0.61	3.48E-29	38744	EZR	−0.53	1.62E-37	5287
NEFM	0.36	7.93E-29	10914	ANXA2P2	−0.46	1.65E-36	6815
GPRC5C	0.63	1.63E-27	2697	TUBB6	−0.67	4.40E-36	3186
SLC20A1	0.61	2.59E-27	2898	LUM	−0.43	4.85E-36	7943
HIST1H2BC	0.64	8.01E-27	2606	THBS1	−0.81	4.40E-34	2192
UBB	0.23	1.36E-26	34818	MYL12A	−0.41	5.11E-33	8065
PHGDH	0.69	3.28E-26	2207	RCAN1	−0.70	2.15E-31	2575
HMGA1	0.39	2.87E-23	5911	PEA15	−0.29	4.72E-31	16083
TMEM158	1.31	7.10E-23	552	BEX1	−0.29	4.72E-31	16766
FADS1	0.58	7.08E-22	2568	PRSS23	−0.62	5.40E-31	3233
EIF1	0.31	2.25E-21	9051	MYL12B	−0.34	1.56E-28	10137
INSIG1	0.51	6.47E-20	2930	SLC7A5	−0.45	1.74E-28	6717
VIM	0.21	1.82E-19	40052	PKP2	−0.89	4.67E-28	1442
SOD2	0.51	1.94E-19	2934	CCND2	−0.60	5.33E-28	3115
NDRG4	0.78	3.03E-19	1248	CPA4	−0.69	1.41E-27	2341
DUSP6	0.87	4.73E-19	1007	FSTL3	−1.01	5.51E-27	1091
CD63	0.20	5.85E-19	35083	UGP2	−0.55	1.10E-25	3325
FTH1	0.23	6.61E-19	94844	TUBB2A	−0.38	2.54E-25	8043
H1FX	0.46	7.92E-19	3449	CNN3	−0.39	7.70E-25	6475
BST2	0.37	1.06E-18	5356	KRT8	−0.36	1.11E-24	36727
RPS11	0.22	1.39E-18	33764	COL11A1	−0.48	1.70E-24	4241
EEF1A2	0.46	4.13E-18	3263	CSRP2	−0.39	5.19E-24	6231
CRABP1	0.65	6.24E-18	1671	GABARAPL1	−0.30	1.03E-23	10528
RGS4	0.65	1.18E-17	1648	PTRF	−0.52	1.38E-23	3487
DDIT3	0.68	1.18E-17	1480	AMOTL2	−0.85	5.17E-23	1275
MVD	0.39	4.14E-17	4372	IVNS1ABP	−0.50	8.57E-23	3556

A) Top 50 genes with higher expression, B) top 50 genes with lower expression

**Table 3:** CMap output of drugs that potentially can be repurposed to treat schizophrenia

CMap name	Primary indication	Class	Mean	*n*	Enrichment	*p*
phenazopyridine^a^	UTI	Analgesic	0.55	4	0.86	0.00056
piperacetazine^b^	Schizophrenia	Antipsychotic	0.56	4	0.85	0.00084
loperamide^c^	Diarrhoea	Opioid receptor agonist	0.32	6	0.74	0.00085
thapsigargin^d^	N/A	Ca-ATPase inhibitor	0.50	3	0.92	0.0011
ampicillin^a^	Infection	Antibiotic	0.45	4	0.84	0.0011
fendiline^c^	Arrythmia	Ca channel blocker	0.54	3	0.91	0.0016
gabapentin^b^	Seizures	Ca channel blocker	0.41	4	0.82	0.0018
monensin^d^	Infection	Antibiotic	0.45	6	0.70	0.0018
niclosamide^a^	Tapeworm	Glucose uptake inhibitor	0.36	5	0.74	0.0029
perphenazine^b^	Schizophrenia	Antipsychotic	0.38	5	0.73	0.0030
ifenprodil^b^	Ptsd	NMDA receptor inhibitor	0.38	4	0.79	0.0036
oxetacaine^a^	GI pain	Local anaesthetic	0.44	5	0.72	0.0039
ciclosporin^c^	Arthritis	Immunosuppressant	0.38	6	0.66	0.0047
cephaeline^c^	Poisoning	Emetic	0.42	5	0.70	0.0064
chlorprothixene^b^	Schizophrenia	Antipsychotic	0.48	4	0.75	0.0070
pimozide^b^	Schizophrenia	Antipsychotic	0.31	4	0.74	0.0080
atracurium besilate^e^	Surgery	Muscle relaxant	0.47	3	0.84	0.0084
alexidine^a^	Infection	Antibiotic	0.29	4	0.74	0.0087
anisomycin^a^	Infection	Antibiotic	0.37	4	0.72	0.012
bepridil^c^	Angina	Ca channel blocker	0.50	4	0.72	0.012
metergoline	Anxiety	DA and 5HT agonist	0.30	4	0.72	0.012
thioproperazine^b^	Schizophrenia	Antipsychotic	0.41	5	0.66	0.012
nifenazone^c^	Arthritis	Analgesic	0.35	5	0.65	0.014
fluoxetine^b^	Depression	Serotonin reuptake inhibitor	0.35	4	0.70	0.017
ifosfamide^c^	Cancer	Alkylating agent	0.37	3	0.79	0.018
gossypol^d^	Antimalarial	Plant phenol	0.24	6	0.58	0.020
nortriptyline^b^	Depression	Tricyclic AD	0.25	4	0.68	0.021
levomepromazine^b^	Palliative care	Antipsychotic	0.28	4	0.68	0.021
raloxifene^b^	Osteoporosis	Estrogen receptor modulator	0.33	7	0.53	0.022
lovastatin^b^	Hypercholesterolemia	HMGCoA reductase inhibitor	0.23	4	0.68	0.022
lomustine^c^	Cancer	Alkylating agent	0.40	4	0.66	0.030
disulfiram	Alcoholism	Acetaldehyde dehydrogenase inhibitor	0.34	5	0.60	0.032
adiphenine^c^	Muscle spasms	Spasmolytic agent	0.37	5	0.60	0.032
naftifine^e^	Tinea	Antifungal	0.19	4	0.64	0.039
semustine^c^	Cancer	Alkylating agent	0.28	4	0.63	0.046
alpha-ergocryptine^d^	N/A	Natural product	0.30	6	0.52	0.047
isotretinoin	Acne	Retinoic acid	0.38	4	0.63	0.047
calycanthine	N/A	Natural product	0.37	4	0.63	0.048
proadifen	N/A	Cytochrome P450 inhibitor	0.26	4	0.63	0.049

^a^Unsuitable for long term use

^b^Not novel

^c^Unacceptable side effect profile

^d^Not approved for use in humans

^e^Poor oral bioavailability

### Locomotor Hyperactivity in Mice

**Fig. 1:**
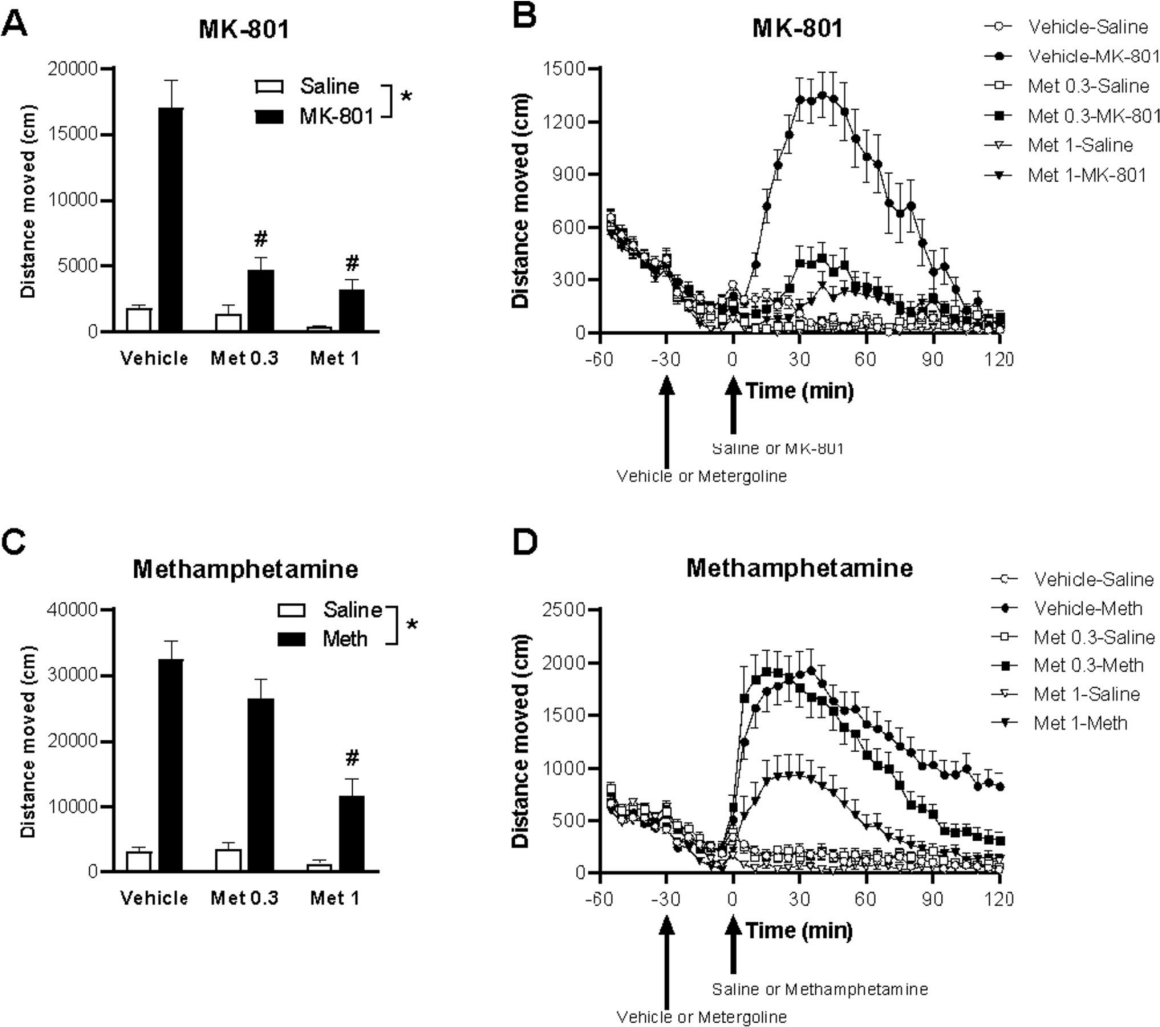
MK-801 or methamphetamine-induced locomotor hyperactivity following administration of vehicle, 0.3 mg/kg metergoline (Met 0.3) or 1 mg/kg metergoline (Met 1). **A** Total distance moved over 2 h immediately following saline or MK-801 injection with vehicle or metergoline injection 30 min prior. *Main effect of MK-801 (p < 0.05) and #post hoc difference to vehicle (p’s < 0.05). **B** The distance moved for each 5-min bin over the full 3-h. **C** Total distance moved over 2 h immediately following saline or methamphetamine (Meth) injection with vehicle or metergoline injection 30 min prior. *Main effect of methamphetamine (p < 0.05) and #post hoc differences to 0.3 mg/kg metergoline and vehicle (p’s < 0.05). **D** The distance moved for each 5-min bin over the full 3-h

Locomotor hyperactivity was induced in mice using either MK-801 or methamphetamine. Repeated measures ANOVA of the total distance moved by mice treated with saline or MK-801 (Fig. 1A) showed a significant main effect of induction, metergoline treatment, and induction × treatment interaction (*p*’s < 0.0001). There was also a significant induction × sex interaction with males showing higher activity in response to MK-801; however, there were no other significant main effects or interactions involving sex for this assessment; therefore, data presented here are pooled across sexes. Post hoc Tukey’s test showed that for saline sessions, there were no significant differences between any treatment (lowest *p* = 0.48), suggesting no effects of metergoline on baseline locomotor activity. For MK-801 sessions, both doses of metergoline significantly reduced MK-801-induced hyperactivity, with a significant difference between vehicle and 0.3 mg/kg metergoline (*p* < 0.0001) and 1 mg/kg metergoline (*p* < 0.0001). The two doses of metergoline did not differ (*p* = 0.45). These results show that metergoline markedly attenuates MK-801-induced locomotor hyperactivity (Fig. 1B)

Repeated measures ANOVA of the methamphetamine-induced locomotor hyperactivity assessment (Fig. 1C) also showed significant main effects of induction and metergoline treatment, as well as an induction × treatment interaction (*p*’s < 0.0001). There was a significant main effect of sex, with females showing overall higher activity than males; however, there were no other significant main effects or interactions involving sex for this assessment, therefore the data presented here are pooled across sexes. Post hoc Tukey’s multiple comparisons showed that there were no significant differences between any treatment (lowest *p* = 0.65) for saline sessions, suggesting again there was no effect of metergoline on baseline locomotor activity. For methamphetamine sessions, 1 mg/kg metergoline significantly reduced methamphetamine-induced locomotor hyperactivity compared to vehicle and 0.3 mg/kg metergoline (both *p* < 0.0001). There was a trend for 0.3 mg/kg metergoline to reduce hyperactivity compared to vehicle (*p* < 0.0573). These results show that metergoline reduces methamphetamine-induced locomotor hyperactivity in a dose-dependent manner (Fig. 1D).

### Confirmation in Mice: Prepulse Inhibition

**Fig. 2:**
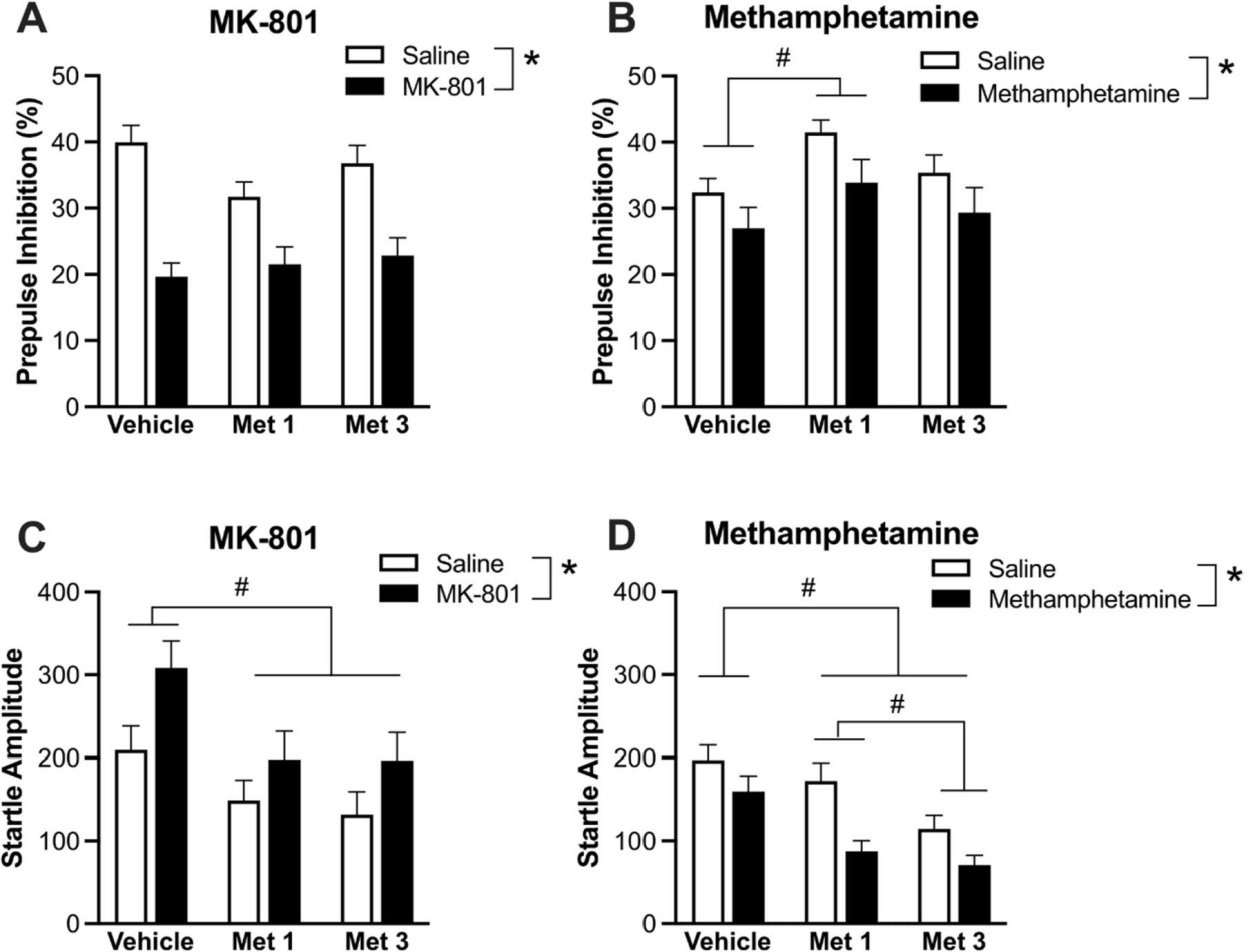
**A** MK-801 or **B** methamphetamine-induced PPI disruption following administration of vehicle, 1 mg/kg metergoline (Met 1) or 3 mg/kg metergoline (Met 3). Startle amplitude following **C** MK-801 or **D** methamphetamine post-administration of vehicle, 1 mg/kg metergoline (Met 1) or 3 mg/kg metergoline (Met 3). *Main effect of MK-801 or methamphetamine (p’s < 0.05), and #post hoc difference between 1 mg/kg metergoline and vehicle (p < 0.05)

For MK-801, repeated measures ANOVA showed a significant main effect of induction and MK-801 treatment (*p* < 0.0001; Fig. 2A), reflecting the expected MK-801-induced decrease of PPI. There was no significant effect of metergoline treatment (*p* = 0.26) or any induction × treatment interaction (*p* = 0.059). There were no significant effects/interactions involving sex. These results show that, unlike the effects on MK-801-induced locomotor hyperactivity, metergoline has no significant effect on MK-801-induced PPI disruption (Fig. 2A).

For methamphetamine, repeated measures ANOVA showed significant main effects of induction and metergoline treatment (both *p* < 0.05), but no induction × treatment interaction (*p* = 0.90). Again, there were no significant main effects or interactions with sex. The main effect of treatment was followed up with post hoc Tukey’s multiple comparisons, which showed 1 mg/kg metergoline significantly increased PPI compared to the vehicle regardless of saline or methamphetamine induction (*p* = 0.035). There were no other significant post-hoc effects (lowest *p* = 0.21). These results show that, unlike the effects on methamphetamine-induced locomotor hyperactivity, metergoline has no significant effect on methamphetamine-induced PPI disruption. Metergoline at 1 mg/kg enhanced PPI, regardless of methamphetamine-induced disruption (Fig. 2B).

Repeated measures ANOVA of startle amplitude following MK-801 (Fig. 2C) or methamphetamine (Fig. 2D) yielded significant main effects of these drugs and of metergoline treatment (both *p* < 0.0001), but no interactions (lowest *p* = 0.062). There were no sex main effects or interactions. These results indicate that MK-801 increased and methamphetamine reduced startle response. The main effects of treatment for each assessment were followed up with post hoc Tukey’s multiple comparisons, which showed both doses of metergoline reduced average startle compared to vehicle (both *p* < 0.001). This effect was dose-dependent for the methamphetamine assessment with startle following 3 mg/kg metergoline treatment significantly reduced compared with 1 mg/kg metergoline (Fig. 2D).

## Discussion

Human neuronal NT2-N cells were treated with a combination of currently prescribed atypical antipsychotic drugs to generate a differential gene expression signature which was used to identify connections between drugs, genes and disease using the CMap database. Genes with increased expression were enriched for circadian rhythm, vascular endothelial growth factor (VEGF) signalling and long-term potentiation pathways. It is widely accepted that disrupted circadian rhythms may play a role in the pathogenesis of schizophrenia (reviewed by Kirlioglu and Balcioglu (2020)), and modulation of circadian factors is a target of current antipsychotics (reviewed by Moon et al. (2021)). There is some evidence to support altered VEGF signalling in schizophrenia (Chu et al. 2009; Frydecka et al. 2018), and one study showed that antipsychotic drugs (haloperidol and olanzapine) increased VEGF signalling in cultured neuronal cells (Jóźwiak-Bębenista et al. 2018). Genes with lower expression following treatment with the antipsychotic drug combination were enriched for several pathways including adherens junction and cell cycle. The adherens junction pathway has been linked to schizophrenia through genetic studies (Yu et al. 2014; Yoon et al. 2014; Hawi et al. 2018), while a breakdown of adherens junctions and increased intestinal permeability have been implicated in the pathophysiology of the disease (Maes et al. 2019), and has been suggested as a new drug target for schizophrenia (Maes et al. 2019, 2021). The link between the adherens junction pathway and synaptic plasticity (Kosik et al. 2005) makes it an interesting target for further investigation. Although the cell cycle pathway has been linked with schizophrenia and the actions of antipsychotic drugs (Benes 2011; Hendouei et al. 2019), and may be linked with mitochondrial dysfunction (Hendouei et al. 2019), the pathway to drug development targeting this pathway is unclear at this time.

The pathways highlighted above may represent new targets for the development of drugs to treat schizophrenia. However, much work is required to determine the suitability of these targets, and even if successful, effective translation is likely many years away. An alternative approach is to repurpose existing drugs. The CMap output highlighted multiple psychoactive drugs as acting similarly to the antipsychotic drug combination used in this study at the transcriptional level, including antipsychotics (piperacetazine, perphenazine, chlorproxithene, pimozide and thioproperazine) and the NMDA receptor antagonist ifenprodil. The analysis also uncovered raloxifene which we have previously shown to have efficacy in schizophrenia (Kulkarni et al. 2016), lovastatin supported by a positive pilot study (Ghanizadeh et al. 2014) and fluoxetine supported by a meta-analysis (Singh et al. 2010). These data therefore confirm the utility of CMap in identifying drugs that have the potential for the treatment of schizophrenia. This approach identified other multiple known antipsychotic drugs, showing construct validity. After excluding drugs with significant barriers to widespread use, the process also highlighted metergoline as a drug with the potential for repurposing to treat neuropsychiatric disorders based on the genetic pathways differentially expressed in neuronal cells treated with a combination of atypical antipsychotic drugs currently used to treat schizophrenia and other neuropsychiatric disorders.

It is crucial to note the distinctions between typical and atypical antipsychotic medications when discussing our results. Typical antipsychotics, also known as first-generation antipsychotics, primarily target dopamine D2 receptors and are more likely to produce extrapyramidal side effects. On the other hand, atypical or second-generation antipsychotics show a more varied receptor profile, including affinity for serotonin receptors, and tend to have a lower risk of such side effects. Given that metergoline exhibits overlapping mechanisms with the serotonergic profile of other atypical antipsychotics, our findings may be particularly pertinent to the development and optimization of second-generation antipsychotic therapies.

Metergoline is currently used to treat seasonal affective disorder (Pjrek et al. 2005; Turner et al. 2002), prolactin hormone regulation (due to its inhibitory effect on prolactin release (Pontiroli et al. 1980)) and premenstrual dysphoric disorder in women (Roca et al. 2002). Metergoline has been shown to have anxiolytic effects in mice (Pellow et al. 1987; Cummings et al. 2014). However, it worsened experimentally induced feelings of anxiety in healthy volunteer humans (Ben-Zion et al. 1999). Metergoline is an ergot-derived psychotropic drug that acts as an antagonist at various serotonin receptor subtypes at relatively low concentrations with overlapping mechanisms with the serotonergic profile of other atypical antipsychotics (Cummings et al. 2014). Typical antipsychotic drugs are strong dopamine D2 receptor antagonists and metergoline also acts as an agonist at dopamine receptors and has effects on various calcium channels (Yeom and Lee 2016).

Extracellular concentration of dopamine is associated with positive psychotic symptoms such as delusions (Guillin et al. 2007; Laruelle et al. 2005), and many antipsychotic drugs have an affinity for subcortical dopamine receptors (Howes et al. 2015). However, dopamine signalling does not fully explain other symptoms such as negative symptoms and cognitive disturbances (Kantrowitz and Javitt 2010). Glutamatergic models of schizophrenia are based on the observation that agents such as phencyclidine and ketamine can induce positive, negative and neurocognitive symptoms of schizophrenia by blocking NMDA-type glutamate receptor signalling (Adell 2020; Haaf et al. 2018). Taken together, combined dysfunction of the glutamate and dopamine systems has been suggested in schizophrenia. Dopamine neuron activity is regulated by glutamatergic projections to the midbrain (Howes et al. 2015) and NMDA-dopamine interactions in mesocorticolimbic regions are well documented (Dallérac et al. 2021; Tseng and O’Donnell 2004). In other words, dopamine disturbances seen in some neuropsychiatric disorders such as schizophrenia could be “downstream” effects of altered glutamatergic function.

We used a behavioural animal model of positive symptoms of schizophrenia, focussing on locomotor hyperactivity and disruptions of PPI, to investigate the effects of metergoline in vivo (van den Buuse 2010). MK-801, also known as dizocilpine, is an NMDA antagonist and therefore affects glutaminergic signalling, and it induced locomotor hyperactivity in mice in this study. This is thought to have some face and construct validity for positive symptoms of schizophrenia, such as psychotic agitation (Powell et al. 2009). Methamphetamine is a well-known indirect dopamine agonist, and it also increased locomotion as expected. PPI is conceptualised as a precognitive process to prevent sensory overload and cognitive fragmentation, and there is evidence that reduced PPI is related to schizophrenia, although it is not a marker specific for the illness (Braff 1993). Reduced PPI is thought to reflect an alteration in dopaminergic and glutamatergic neurotransmission and in preclinical models to reflect both positive and cognitive symptoms (Braff 1993). In the present study, MK-801 and methamphetamine disrupted PPI as expected (van den Buuse 2010; Kraeuter et al. 2020).

We show for the first time that metergoline significantly alleviates MK-801- and methamphetamine-induced locomotor hyperactivity, behaviours relevant to schizophrenia symptoms. Importantly, these effects were not associated with significantly reduced baseline locomotor activity. Our findings are consistent with previous studies showing 1 mg/kg metergoline reduced dizocilpine-induced hyperactivity in male rats (Varty and Higgins 1995; Nanry and Tilson 1989), supporting metergoline as a potential candidate to be repurposed to treat schizophrenia or other neuropsychiatric disorders. In other studies, metergoline’s effects on behaviours relevant to schizophrenia have been mainly investigated using PPI (Varty and Higgins 1995). Metergoline injected at 0.25 mg/kg in male rats has been shown to enhance PPI but this effect was attributed to changes in baseline startle. Interestingly, we also observed enhanced PPI due to 1 mg/kg metergoline, although this effect was not related to methamphetamine-related PPI deficits. This effect was furthermore not replicated in the mice used for MK-801-induced PPI disruption. Another study in male rats showed that 1 mg/kg metergoline reversed a dizocilpine-induced PPI deficit (Varty and Higgins 1995) although our results showed no such effect. This apparent discrepancy may be explained by differences in neural substrates and neuropsychopharmacology mechanisms in the regulation of PPI between mouse lines and mice vs rats where MK-801 may mediate PPI disruption by different mechanisms (Bakshi and Geyer 1998). Other studies have similarly shown differences between rats and mice in the regulation of PPI. For example, injection of a dopamine receptor agonist directly into the nucleus accumbens increased PPI in mice (Mohr et al. 2007) but decreased it in rats (Wan et al. 1995). Systemic treatment with a serotonin-1A receptor agonist decreased PPI in rats (Gogos et al. 2005) but increased it in mice (Gogos et al. 2008). In mice, dopamine D1 receptors have a more prominent role in the regulation of PPI than D2 receptors, whereas in rats it is the reverse (Ralph-Williams et al. 2003). Such findings on species differences warrant future studies in rats to further investigate the effects of metergoline on PPI regulation.

The limitations of this study pertain primarily to the scope of the behavioural assays employed. While our focus on locomotor hyperactivity and PPI is aligned with traditional methodologies in the field, we recognize that the inclusion of further assays like latent inhibition, social interaction, and cognitive function tests could offer a more nuanced understanding of metergoline’s therapeutic potential across a broader spectrum of neuropsychiatric symptoms (Jones et al. 2011; Gobira et al. 2013; Nikiforuk 2018).

A specific consideration when utilizing traditional behavioural tests is the propensity to identify compounds that bear a strong resemblance to pre-existing treatments, both mechanistically and phenotypically. This may inadvertently restrict the innovation space for the discovery of fundamentally new treatments. Nonetheless, the very act of repurposing approved drugs, as pursued in this study, offers a streamlined path to human clinical trials due to pre-existing safety and usage data.

## Conclusion

In conclusion, we identified metergoline as a candidate drug with a similar potential effect on genetic pathways altered in human neuronal cells treated with a combination of atypical antipsychotic drugs. Our findings suggest that metergoline has the potential to reduce drug-induced locomotor hyperactivity in mice, as an indirect approach to test its potential effect in the treatment of positive symptoms of schizophrenia. Further studies will need to be addressed to fully explore the effect of metergoline in psychiatric disorders. However, our results highlight metergoline as a promising candidate drug to be repurposed for further studies as effective treatment for neuropsychiatric disorders.

## Data Availability

The datasets generated and/or analysed in this study are available from the corresponding author upon reasonable request. The materials used in this study, including any software, scripts, or protocols, are also available upon request to the corresponding author.
